# Self‐Esteem and Coping Modes as Mediators of the Social Support‐Stigma Relationship in Rheumatoid Arthritis Patients: A Multicentre Cross‐Sectional Study

**DOI:** 10.1002/nop2.70630

**Published:** 2026-06-21

**Authors:** Yi Zhang, Bo Gao, Yimin Ma, Xiaoying Dai, Lifeng He, Yaqin Geng

**Affiliations:** ^1^ Department of Rheumatology The Third Affiliated Hospital of Nanjing Medical University Changzhou China; ^2^ Department of Nursing Nanjing Medical University Nanjing China; ^3^ Department of Rheumatology Taixing People's Hospital Taixing China; ^4^ Department of Rheumatology The First Affiliated Hospital of Wannan Medical College Wuhu China

**Keywords:** coping modes, nursing, rheumatoid arthritis, self‐esteem, social support, stigma

## Abstract

**Aim:**

To explore the chain mediating effects of self‐esteem and coping modes on the relationship between social support and stigma with rheumatoid arthritis patients and to identify actionable targets for nursing interventions.

**Design:**

A multicentre, cross‐sectional, correlational study.

**Methods:**

Using convenience sampling, RA patients were recruited from the rheumatology departments of four hospitals in China, from June to September 2023. The Internalized Stigma of Mental Illness Scale‐Rheumatoid Arthritis (ISMI‐RA), Social Support Rating Scale (SSRS), Rosenberg Self‐Esteem Scale (RSES) and Medical Coping modes *Q*uestionnaire (MCMQ) were used to assess the patients' stigma, social support, self‐esteem and coping styles. SAS 9.4 software was used for statistical analysis. Normally distributed continuous data were described using means and standard deviations. Independent sample *t*‐tests and analysis of variance (ANOVA) were used for comparisons between groups. The Pearson correlation coefficient was used to describe the degree of correlation between variables. The chain mediating model was constructed using AMOS 24.0 software, and the maximum likelihood method was used for model parameter estimation, and the model was adjusted according to the modification index.

**Results:**

Mean participant age was 53.62 ± 12.40 years. Stigma scores differed significantly by family history, income, education, morning stiffness duration, DAS28 and pain level (all *p* < 0.001). Stigma correlated negatively with self‐esteem (*r* = −0.482), confrontation coping (*r* = −0.512) and social support (*r* = −0.590) and positively with avoidance (*r* = 0.251) and acceptance coping (*r* = 0.462). Social support was directly associated with stigma (*β* = −0.502) and also indirectly associated through self‐esteem and coping modes, with the indirect pathways accounting for 31.51% of the total association.

**Conclusion:**

The findings suggest that patients with RA frequently experience moderate stigma, and that self‐esteem and coping modes may serve as chain mediators between social support and stigma. Nurses may consider assisting patients in building positive social support networks, enhancing their self‐esteem and promoting positive coping modes as potential strategies to reduce stigma.

## 1 Introduction

Rheumatoid arthritis (RA) is a common autoimmune disease, characterised by erosive arthritis. Epidemiological surveys indicate a worldwide prevalence of 0.5% to 1% of RA. Moreover, the global prevalence has surged by 7.4% from 1999 to 2017, accompanied by an 8.2% increase in its incidence (Finckh et al. 2022). In China, the prevalence rate ranges from 0.28% to 0.41%, with over 5 million individuals affected by the disease, imposing a significant burden on society (Tian et al. 2021). Rheumatoid arthritis causes joint damage and disability that impairs physical function and negatively affects mental well‐being (Zhou et al. 2018). This impairs daily living, work (Syngle et al. 2020) and social functioning (Poh et al. 2017), while lack of public understanding leaves patients feeling disregarded, further contributing to negative emotions and stigma (Tiwana et al. 2015).

Medically, stigma refers to the internalised feelings of shame experienced by patients because of their disease, possibly leading to psychological disorders, diminished self‐esteem, reduced willingness to seek treatment and in extreme cases, suicide (Ociskova et al. 2023). Individuals with RA often face limitations in their professional and social engagements due to symptoms such as fatigue, pain and disability, thereby increasing the risk of unemployment and social exclusion (Bay et al. 2020), which may further exacerbate stigma. Previous research has shown that the level of social support provided to patients is associated with their experience of stigma (Karaçar and Bademli 2022). When patients receive appropriate support and care from society, they often find it easier to manage life's difficulties and challenges, which may reduce negative emotions. Although previous studies have extensively investigated the stigma associated with conditions such as AIDS (Wang et al. 2023), mental illness (Mitelman et al. 2023) and diabetes (Luo et al. 2023), studies on the stigma experienced by patients with RA have received little attention.

Self‐esteem is a positive psychological state and emotional attitude encompassing the ability to recognise and identify with oneself, as well as confidence in one's abilities. The level of personal self‐esteem may affect an individual's perception and assessment of their behaviours. Individuals with low self‐esteem may experience cognitive biases related to underlying beliefs, causing a greater tendency to feel threatened about their value and generate negative evaluations. These evaluations may activate underlying negative beliefs, triggering negative emotions and stigma (Rimes et al. 2023). Rosenberg proposed that self‐esteem possesses social attributes and is affected not only by individual internal factors but also by the social environment (AlHarbi 2022). Monsonet reported a positive correlation between the intensity and quality of social interactions with self‐esteem (Monsonet et al. 2023). Similarly, Harris's study revealed that there is a positive correlation between social support and self‐esteem (Harris and Orth 2020). Social support provides individuals with emotional support, affirmation and encouragement, allowing them to feel cared for and recognised by others, which in turn enhances self‐esteem.

Individuals' coping modes may be associated with stigma. The coping modes employed by individuals may contribute to the generation and management of stigma (Yang et al. 2022). A study reported that adopting a positive coping modes was associated with individuals' acceptance and adjustment to health concerns, thereby potentially mitigating the adverse effects of stigma (Niluopa and Rena 2023). Furthermore, social support may be associated with an individual's coping modes, as good social support may enhance an individual's sense of security and self‐worth, thereby broadening the psychological buffer zone (Hong et al. 2023; Sharp et al. 2023). These studies suggest that stigma may be associated with social support, self‐esteem and coping modes. However, research focusing on the relationships and internal mechanisms among these four factors is scarce, particularly from a nursing practice perspective. Understanding these mechanisms is essential for developing evidence‐based nursing interventions. Therefore, these relationships warrant further investigation. This study hypothesises that self‐esteem and coping modes function as mediators in the relationship between social support and stigma. The findings are intended to guide nurses in designing holistic, patient‐centred rheumatology care plans to address the psychological burden of RA.

## 2 Methods

### 2.1 Study Design and Rationale

This study employed a multicentre, cross‐sectional, correlational design. A cross‐sectional design was chosen to achieve the following aims appropriate for an exploratory nursing study: (1) to estimate the prevalence and magnitude of stigma and its key correlates (self‐esteem, coping, social support) in a naturalistic clinical setting, which is essential for hypothesis generation; (2) to examine the chain mediating pathways among these four psychosocial variables, providing initial evidence of the direction and structure of associations that can later be tested in longitudinal nursing intervention studies; and (3) to efficiently recruit a reasonably sized, clinically accessible sample from multiple hospital sites, enhancing the generalisability of findings to routine rheumatology nursing practice. While causal inference is not possible, this design is the standard for establishing mediation pathways as a prerequisite for future experimental nursing research.

### 2.2 Participant and Procedure

We recruited patients from the rheumatology outpatient departments of four tertiary hospitals in China between June and September 2023. Inclusion criteria were: (1) age ≥ 18 years; (2) meeting the 2010 ACR/EULAR classification criteria for RA (Wasserman 2011); (3) normal cognitive function and ability to communicate verbally or in writing; (4) willingness to provide informed consent. Exclusion criteria were: (1) coexisting other rheumatic autoimmune diseases (e.g., systemic lupus erythematosus, psoriatic arthritis); (2) diagnosed mental illness (e.g., major depression, schizophrenia) prior to RA diagnosis; (3) active malignancy or other serious comorbidities (e.g., end‐stage renal disease, heart failure) that could independently influence psychological status; (4) participation in another psychosocial intervention study concurrently.

Eligible patients were approached consecutively during scheduled rheumatology visits. A trained research nurse explained the study purpose, assured confidentiality and obtained written informed consent. Self‐report questionnaires were completed in a private room at the clinic, with the research nurse available to clarify items but not to influence responses. For patients with literacy difficulties, the nurse read items aloud neutrally.

### 2.3 Sample Size Calculation

Sample size was determined considering three factors. First, the analysis included up to 23 independent variables (12 demographic/disease variables + 11 scale dimensions). With a recommended 5–10 participants per variable and allowing for 10% incomplete data, the minimum required sample was 127 to 253. Second, structural equation modelling (SEM) for chain mediation typically requires ≥ 200 participants to achieve stable parameter estimates and adequate statistical power (Wu, n.d.). Third, to account for potential missing data and non‐normal distributions, we conservatively targeted 220 participants. This sample size exceeds the minimum for SEM with 5000 bootstrap resamples.

### 2.4 Measures

Demographic characteristics contained sex, age, family history, marital status, occupational status, monthly personal income, education level, medical payment methods. Disease‐related characteristics included the duration of morning stiffness, disease course, pain level, Disease Activity Score in 28 joints score.

Internalized Stigma of Mental Illness Scale for Rheumatoid Arthritis (ISMI‐RA) was used to assess stigma in patients with rheumatoid arthritis. The scale has 20 items, including four dimensions, with scores ranging from 20 to 80. This scale uses a Likert 4‐level scoring method, with each item having a possible score from 1 to 4 corresponding to the responses ‘strongly disagree,’ ‘disagree,’ ‘agree,’ and ‘strongly agree,’ with a total score ranging from 20 to 80. Higher scores on this scale represent higher levels of stigma intensity. The Cronbach's *α* coefficient is 0.86 (Corker et al. 2016). The Chinese version of the scale has been validated in patients with Rheumatoid Arthritis, demonstrating good reliability and validity.

Self‐esteem was measured using the Rosenberg Self‐Esteem Scale (RSES). The scale comprises 10 items with scores ranging from 10 to 40. For the forward scoring items, item scores range from 1 (very nonconforming) to 4 (very conforming). Item 3, 5, 9 and 10 were reverse‐scored, with item scores ranging from 1 (very conforming) to 4 (very nonconforming). Higher scores on the RSES indicate higher levels of self‐esteem. This scale is widely used nationally and internationally, exhibiting good reliability and validity. The Chinese version of RSES has shown good psychometric properties in various Chinese populations, with reported Cronbach's *α* coefficients typically above 0.80 (Chung et al. 2021).

Coping modes were analyzed using the Medical Coping modes Questionnaire (MCMQ) (Tian et al. 2009). The questionnaire consists of three dimensions: avoidance, acceptance and confrontation, amounting to 20 items. Each item is scored from 1 to 4 points according to the Likert 4‐level scoring method, including eight reverse‐scored items. Higher scores in a particular dimension suggest that the patient has a greater tendency to cope with that specific mode. The scores for avoidance, acceptance and confrontation ranged from 8 to 32, 7 to 28 and 5 to 20, respectively. Cronbach's *α* coefficient for the avoidance, acceptance and confrontation dimensions was 0.60, 0.76 and 0.69, respectively (Feifel et al. 1987).

Social support was assessed using Social Support Rating Scale (SSRS) (Xiao 1994). The SSRS is a 10‐item scale with domains comprising objective support, subjective support and utilisation of support. As for the scoring of the SSRS, items 1–4 and 8–10 allow the selection of only one response, with the responses numbered 1, 2, 3 and 4 corresponding to 1, 2, 3 and 4 points, respectively. Item 5 contains four options (A, B, C and D), wherein more than one option can be selected. The responses for each option in item 5 are scored from 1 (no support) to 4 points (full support). Items 6 and 7 are scored 0 points if the participants select the ‘no source’ response. Alternatively, they can respond with one or several options under the ‘the following source’ response, with each option counted as 1 point. The total score of the SSRS is the sum of the scores of the 10 items. Additionally, the objective support score is the combined scores of items 2, 6 and 7; the subjective support score, items 1, 3, 4 and 5; and the utilisation of support score, items 8, 9 and 10. Higher scores on the SSRS denote greater social support. This scale has good reliability and validity, with Cronbach's α coefficient ranging from 0.89 to 0.91 (Ye and Dai 2008).

### 2.5 Data Analysis

SAS software (version 9.4; SAS Institute Inc., Cary, NC, USA) was used for descriptive statistics, group comparisons and correlation analyses. Normally distributed continuous data were described using means and standard deviations. Between‐group comparisons were conducted using independent‐sample *t*‐tests (two groups) and one‐way ANOVA (three or more groups), with post hoc pairwise comparisons performed using the Student–Newman–Keuls (SNK‐q) test. Pearson correlation coefficients were used to describe bivariate associations between continuous variables.

AMOS software (version 24.0; IBM Corp., Armonk, NY, USA) was used to construct the chain mediation model. Maximum likelihood estimation was used for model parameter estimation. The model was specified with social support as the exogenous (independent) variable, stigma as the endogenous (dependent) variable and self‐esteem and coping modes (confrontation, avoidance, acceptance) as sequential mediators. Model fit was evaluated using the following thresholds: *χ* ^2^/df < 5.0, RMSEA < 0.08, GFI ≥ 0.90, IFI ≥ 0.90 and CFI ≥ 0.90. Model adjustments were made iteratively based on modification indices, but only when theoretically justified (e.g., allowing correlated error terms between conceptually related items). The bias‐corrected percentile bootstrap method with 5000 resamples was used to calculate 95% confidence intervals for all indirect (mediated) effects. A 95% CI that did not include zero was considered statistically significant for mediation. The significance level for all other analyses was set at *α* = 0.05 (two‐tailed).

## 3 Results

### 3.1 Descriptive Statistics

The 220 participants (86.36% female) had a mean age of 53.62 years (SD = 12.40). Mean disease duration was 9.03 years (SD = 8.07); 51.36% had moderate disease activity. Mean stigma score was 52.25 (SD = 10.41) (Table 1).

**TABLE 1:** Descriptive statistics of sociodemographic and scale scores (*N* = 220).

	*n* (%)	*M* (SD)	Range
Sex			
Male	30 (13.64)		
Female	190 (86.36)		
Age (year)		53.62 (12.40)	24–87
18–44	41 (18.63)		
45–59	118 (53.64)		
≥ 60	61 (27.73)		
Family history			
Yes	159 (72.27)		
No	61 (27.73)		
Marital status			
Married	214 (97.27)		
Single/Divorced/widowed	6 (2.73)		
Occupational status			
Employed	133 (60.45)		
Unemployed	22 (10.00)		
Retired	65 (29.55)		
Monthly personal income (RMB)			
< 3000	86 (39.09)		
3000–5000	98 (44.55)		
> 5000	36 (16.36)		
Medical payment mode			
Medical insurance	162 (73.64)		
NCMS	41 (18.64)		
Self‐payment	17 (7.73)		
Educational level			
Middle school or below	130 (59.09)		
Technical secondary/high school	57 (25.91)		
Junior college or above	33 (15.00)		
Course of disease (year)		9.03 (8.07)	0.08–36
< 10	150 (68.18)		
≥ 10	70 (31.82)		
Duration of morning stiffness (min)			
≤ 15	155 (70.45)		
16–59	42 (19.09)		
≥ 60	23 (10.45)		
DAS28			
≤ 2.6	46 (20.91)		
2.6–3.2	20 (9.09)		
3.21–5.1	113 (51.36)		
> 5.1	41 (18.64)		
Pain level (VAS)			
0–3	104 (47.27)		
4–6	94 (42.73)		
7–10	22 (10.00)		
ISMI‐RA total		52.25 (10.41)	21–76
Alienation		13.95 (3.11)	5–20
Stereotype endorsement		10.55 (2.33)	4–16
Perceived discrimination		11.90 (2.77)	6–19
Social withdrawal		15.86 (3.22)	6–24
RSES total		28.87 (4.13)	21–37
MCMQ			
Confrontation		21.33 (2.35)	15–26
Avoidance		15.17 (2.09)	10–21
Acceptance		10.87 (2.26)	8–16
SSRS total		41.63 (9.03)	18–63
Subjective support		9.84 (3.73)	3–20
Objective support		24.62 (5.49)	11–32
Support utilisation		7.16 (2.13)	3–12

Abbreviations: DAS28, Disease Activity Score 28; ISMI‐RA, Internalized Stigma of Mental Illness scale‐Rheumatoid Arthritis; *M*, mean; MCMQ, Medical Coping Mode Questionnaire; NCMS, New Cooperative Medical Scheme; RSES, Rosenberg Self‐Esteem Scale; SD, standard deviation; SSRS, Social Support Rating Scale.

Stigma scores differed significantly by family history, income, education, morning stiffness, DAS28 and pain level (all *p* < 0.001). Post hoc tests showed highest stigma in the lowest socioeconomic groups and among those with the most severe clinical presentations (Table 2).

**TABLE 2:** Differences in stigma score by sociodemographic variables (*N* = 220).

		*M*	SD	*F*/*t*	*p*
Family history					
Yes		54.06	9.56	4.326	< 0.001
No		47.54	11.11		
Monthly personal income (RMB)					
< 3000	a	55.94bc	9.58	16.451	< 0.001
3000–5000	b	51.70ac	9.23		
> 5000	c	44.94ab	11.38		
Educational level					
Middle school or below	a	54.89bc	9.61	12.912	< 0.001
Technical secondary/high school	b	49.86ac	9.96		
Junior college or above	c	46.00ab	10.78		
Duration of morning stiffness (min)					
≤ 15	a	50.56c	10.3	10.019	< 0.001
16–59	b	54.24c	8.61		
≥ 60	c	60.04ab	10.3		
DAS28					
≤ 2.6	a	47.26cd	10.93	7.465	< 0.001
2.6–3.2	b	50.15d	10.86		
3.21–5.1	c	52.92a	9.28		
> 5.1	d	57.05ab	10.27		
Pain level (VAS)					
0–3	a	48.83bc	10.2	22.217	< 0.001
4–6	b	53.47ac	9.03		
7–10	c	63.27ab	8.08		

*Note:* Multiple comparisons by SNK‐q test: a, compared with layer a, *p* < 0.05; b, compared with layer b, *p* < 0.05; c, compared with layer c, *p* < 0.05; d, compared with layer d, *p* < 0.05.

### 3.2 Correlations Between RSES, MCMQ, SSRS and ISMI‐RA

As shown in Table 3, stigma was negatively correlated with RSES (*r* = −0.482), MCMQ confrontation (*r* = −0.512) and SSRS (*r* = −0.590). Stigma was positively correlated with MCMQ avoidance (*r* = 0.251) and acceptance (*r* = 0.462) copings.

**TABLE 3:** Correlations (*r*) between RSES, MCMQ, SSRS and ISMI‐RA.

	RSES	Confrontation	Avoidance	Acceptance	SSRS	ISMI‐RA
RSES	1					
MCMQ						
Confrontation	0.227***	1				
Avoidance	−0.175**	−0.047	1			
Acceptance	−0.165*	−0.188**	0.192**	1		
SSRS	0.381**	0.275**	−0.028	−0.310**	1	
ISMI‐RA	−0.482***	−0.512***	0.251***	0.462***	−0.590**	1

Abbreviations: ISMI‐RA, Internalized Stigma of Mental Illness scale‐Rheumatoid Arthritis; MCMQ, Medical Coping Mode Questionnaire; RSES, Rosenberg Self‐Esteem Scale; SSRS, Social Support Rating Scale.

* *p* < 0.05.

** *p* < 0.01.

*** *p* < 0.001.

### 3.3 Mediation Analyses

**FIGURE 1:**
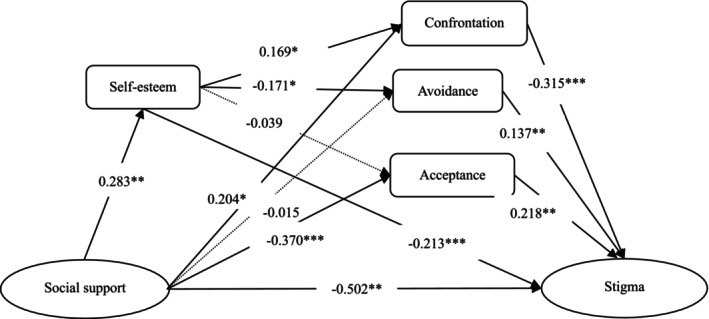
The model of the chained mediating effect of self‐esteem and coping modes between social support and stigma (*p < 0.05; **p < 0.01; ***p < 0.001).

A chain mediation model was constructed with social support as the independent variable, self‐esteem and coping modes as mediators and stigma as the dependent variable (Figure 1). The model showed good fit: *χ* ^2^/df = 2.284, GFI = 0.941, CFI = 0.961, RMSEA = 0.077 (Table 4).

**TABLE 4:** Path coefficients of multiple mediating effect models.

	Estimate (*β*)	95% CI	*p*
Direct effect			
SS → IS	−0.502	−0.642 to −0.360	0.001
Indirect effect			
SS → SE → CF → IS	−0.015	−0.041 to −0.003	0.011
SS → SE → AD → IS	−0.007	−0.020 to −0.001	0.011
SS → SE → AT → IS	−0.002	−0.013 to 0.006	0.422
SS → SE → IS	−0.060	−0.103 to −0.033	< 0.001
SS → CF → IS	−0.064	−0.118 to −0.014	0.018
SS → AD → IS	−0.002	−0.027 to 0.033	0.846
SS → AT → IS	−0.081	−0.138 to −0.042	< 0.001
Total effect			
SS → IS	−0.733	−0.848 to −0.603	< 0.001

Abbreviations: AD, avoidance; AT, acceptance; CF, confrontation; IS, internalised stigma; SE, self‐esteem; SS, social support.

#### 3.3.1 Direct Associations

Social support was directly associated with stigma (*β* = −0.502, 95% CI = −0.642 to −0.360, *p* = 0.001). Self‐esteem was directly associated with stigma (*β* = −0.213, 95% CI = −0.306 to −0.106, *p* < 0.001). Confrontation coping was directly associated with stigma (*β* = −0.315, 95% CI = −0.418 to −0.201, *p* < 0.001), while acceptance coping showed a positive direct association (*β* = 0.218, 95% CI = 0.099 to 0.338, *p* = 0.001).

#### 3.3.2 Indirect Associations

The total indirect effect of social support on stigma through self‐esteem and coping modes was *β* = −0.231 (95% CI = −0.334 to −0.150), accounting for 31.51% of the total association (total *β* = −0.733, 95% CI = −0.848 to −0.603). Specific indirect pathways are summarised as follows:

Social support → self‐esteem → stigma: *β* = −0.060 (95% CI = −0.103 to −0.033).

Social support → confrontation coping → stigma: *β* = −0.064 (95% CI = −0.118 to −0.014).

Social support → acceptance coping → stigma: *β* = −0.081 (95% CI = −0.138 to −0.042).

Social support → self‐esteem → confrontation coping → stigma: *β* = −0.015 (95% CI = −0.041 to −0.003).

Social support → self‐esteem → avoidance coping → stigma: *β* = −0.007 (95% CI = −0.020 to −0.001).

Self‐esteem also showed indirect associations with stigma through confrontation and avoidance coping (total *β* = −0.085, 95% CI = −0.172 to −0.019), accounting for 28.52% of the total association between self‐esteem and stigma. All 95% confidence intervals for indirect effects excluded zero, indicating statistically significant mediated associations.

## 4 Discussion

In the present study, we found that patients with RA commonly experience moderate stigma. The results also suggest that social support, self‐esteem and coping modes are associated with stigma. These findings offer insights into potential interventions aimed at addressing stigma in RA.

In this study, the mean total score for the ISMI‐RA was 52.25 (SD = 10.41), which corresponds to an item mean of 2.61 (SD = 0.52; range: 1.05–3.80), higher than that reported in some foreign studies (Corker et al. 2016). This difference may involve several factors such as social culture, healthcare system and chronic disease education. It is necessary to comprehensively consider these factors and provide appropriate support and assistance to assist patients in better managing and treating diseases. Research results revealed that sociodemographic factors and disease‐related information impact the levels of stigma by patients. Those with a family medical history generally exhibit more intense feelings of stigma. Epidemiological studies have reported a genetic predisposition to RA (Padyukov 2022), which may lead patients to believe that the disease is hereditary and unavoidable, with concerns about passing on the condition to their family members, potentially intensifying stigma. Nurses should understand the specific situations and needs of each patient, encourage them to communicate openly with their family members, help family members understand their difficulties and needs, face the illness together and establish a support system within the family. Furthermore, economic status and educational attainment were linked to the stigma. For instance, individuals with lower incomes tended to experience heightened levels of worry and anxiety when confronted with the substantial costs of medical treatment, fearing the added financial strain on their families and subsequently experiencing feelings of guilt. These findings align with the outcomes of other studies (Zhang et al. 2022). Patients with higher education levels generally have a stronger health awareness and knowledge, helping them better understand and manage health issues. Nurses may use the Transcultural Nursing Theory (McFarland and Wehbe‐Alamah 2019) to provide personalised nursing measures based on patients' different social backgrounds and cultural levels. The morning stiffness duration, degree of pain and DAS28 score reflect RA severity and disease activity. A higher disease activity is associated with more severe symptoms such as joint pain and swelling in patients and a greater impact on daily life. These patients may experience more physical discomfort and fatigue, which may increase emotional distress and stigma. Thus, clinicians should timely and dynamically assess disease activity, treat patients in a stratified manner based on disease activity levels, monitor their physical and mental status and intervene effectively and promptly.

This study also found a negative association between social support and stigma, consistent with other studies (Wu et al. 2023). Social support may contribute to managing stress, strengthening self‐regulation skills and enhancing psychological resilience (Wang et al. 2024). Studies have demonstrated that social support positively influences individuals' health and overall well‐being (Nakagomi et al. 2023). Thus, building a robust social support system may help prevent and alleviate stigma. Research has demonstrated that emotional support from family members is related to reduced negative emotions in patients with RA (Bergström et al. 2023). It is recommended that nurses encourage the involvement of family members in the patient's treatment and rehabilitation process, facilitating their access to familial support and understanding and collaboratively addressing the challenges posed by the illness. Additionally, self‐esteem was negatively related to stigma. Assisting the patients to establish a positive self‐image and encouraging them to accept their uniqueness and limitations can help alleviate negative emotions and self‐denial. Confrontation coping was negatively associated with stigma, while avoidance and acceptance coping showed positive associations with stigma. In this study, the majority of patients are more inclined to use the coping modes of confrontation, but there are still 23.6% of patients who adopt negative coping modes. Clinical practitioners can establish peer support groups, encourage patients with good coping modes to share their life experiences and self‐recovery skills, help other patients reduce anxiety, loneliness and depression, and enhance psychological resilience. It is noteworthy that some of the observed correlation coefficients, particularly between MCMQ avoidance and stigma (*r* = 0.251), had absolute values below 0.3, which might be considered weak in some contexts. However, several factors support the relevance of these findings. First, the study sample was clinically homogeneous, consisting solely of RA patients, which can restrict the range of psychological scores and attenuate correlation magnitudes compared to more heterogeneous populations. Second, within the framework of a complex psychosocial model involving multiple mediating pathways, even modest correlations can contribute meaningfully to the overall chain of effects. The significant role of avoidance coping in the mediation model, despite its weaker bivariate correlation, underscores that its effect may be more pronounced when operating in concert with other variables like self‐esteem rather than in isolation.

Finally, self‐esteem and coping modes showed a chain mediating association between social support and stigma. The results demonstrated that the indirect effects of self‐esteem and coping modes accounted for 31.51% of the total effect of social support on stigma. This is consistent with the idea that social support may be associated with patients' stigma not only directly but also indirectly via self‐esteem and coping modes, offering a new perspective for interventions targeting stigma. Furthermore, patients with higher levels of social support tend to have stronger self‐esteem (Lu et al. 2023) and are more likely to select the positive coping modes. Effective social support can substantially strengthen an individual's sense of self‐identity. When an individual's self‐esteem is safeguarded and acknowledged, they typically employ positive coping modes to address issues and improve their ability to adapt to different changes and challenges. Therefore, enhancing self‐esteem and adopting positive coping modes can be employed as intervention goals to further alleviate feelings of stigma among patients with RA. However, the results of this study indicate that self‐esteem and coping modes only partially mediate the relationship between social support and stigma, suggesting the presence of other variables with mediating effects between them. Further studies are warranted to understand the underlying mechanisms. Furthermore, although this study did not directly measure personality traits, it is essential to acknowledge the fundamental role personality plays in shaping an individual's self‐esteem and coping styles, which may subsequently influence their experience of stigma. Self‐esteem, as a core belief about one's own worth, is profoundly influenced by relatively stable personality traits. For instance, individuals with high levels of Neuroticism may be more prone to lower self‐evaluation and fear of social rejection, thereby exacerbating illness‐related internalised stigma. Conversely, traits such as Conscientiousness and Extraversion are likely associated with more positive self‐perceptions and proactive coping strategies, which may serve a buffering function. A recent study on patients with chronic illnesses emphasised the importance of considering personality factors for a comprehensive understanding of the psychological adaptation process (Cojocaru et al. 2024). Similarly, in the field of rheumatic diseases, personality traits have been demonstrated to be significantly associated with pain perception, illness coping and overall quality of life (Gokcen et al. 2022). Therefore, future research that incorporates personality assessments would help to elucidate the complex internal mechanisms linking social support, self‐esteem, coping patterns and stigma more deeply. This would, in turn, provide a theoretical basis for developing personalised psychological intervention strategies tailored to patients with different personality characteristics.

Based on the findings of this study, several directions for future clinical practice and research are proposed. Firstly, developing and testing psychosocial interventions that simultaneously target the enhancement of social support, the cultivation of self‐esteem and the training of positive coping modes (e.g., confrontation) is crucial. Such multi‐component interventions could include structured family and peer support programmes, cognitive‐behavioural therapy techniques to challenge negative self‐perceptions and skill‐building workshops for adaptive coping strategies. Secondly, future research should employ longitudinal or interventional designs to establish causal relationships among these variables and to evaluate the long‐term effectiveness of these interventions in reducing stigma. Finally, exploring the role of personality traits, as potential antecedents or moderators in this psychological mechanism could provide a more comprehensive understanding and inform highly personalised care strategies for RA patients.

### 4.1 Implications for Nursing Practice

The findings of this study offer several practical directions for rheumatology nursing. First, routine nursing assessment should include not only clinical disease activity (DAS28, pain, morning stiffness) but also psychosocial variables such as perceived social support, self‐esteem and coping styles. Simple screening questions or brief validated tools (e.g., the RSES, a 10‐item scale) can be integrated into intake nursing assessments. Second, nurses can design and implement targeted interventions to reduce stigma. Based on the chain mediation results, interventions that simultaneously strengthen social support and enhance self‐esteem and promote confrontation coping are likely to be more effective than single‐focus approaches. For example:

- Social support enhancement: Facilitate family involvement in education sessions, establish RA peer support groups and provide referrals to community patient organisations.
- Self‐esteem strengthening: Use cognitive reframing techniques to challenge negative self‐perceptions related to disability, celebrate small functional achievements and use positive affirmation statements in nurse–patient interactions.
- Coping skills training: Offer structured group or individual sessions teaching problem‐solving skills, assertive communication and stress management to increase confrontation coping while reducing avoidance/acceptance coping patterns.

### 4.2 Limitations

This study has several limitations. First, the cross‐sectional design precludes causal inference; all associations should be interpreted as correlational rather than causal. Second, all measures were self‐reported, which may introduce common method bias—systematic variance due to the measurement method rather than the constructs of interest—potentially inflating observed associations. Third, convenience sampling from four tertiary hospitals in Jiangsu and Anhui provinces limits generalisability; findings may not be representative of RA patients in other regions, primary care settings or community populations. Fourth, the sample was predominantly female, which may limit the generalisability of findings to male RA patients. Future studies should employ longitudinal designs, diverse and representative samples and objective measures where possible to confirm and extend these findings.

## 5 Conclusions

The findings suggest that patients with RA frequently experience moderate stigma, and that self‐esteem and coping modes may serve as chain mediators between social support and stigma. This highlights the importance for healthcare providers to consider patients' psychological well‐being during treatment, particularly focusing on those with a family history of RA, low income, low education and severe conditions. Nurses should not only attend to patients' social support but also focus on assessing and potentially enhancing their self‐esteem and guiding them in adopting positive coping modes as part of strategies to alleviate stigma.

## Author Contributions

Y.Z. and B.G.: conceptualisation, data curation, investigation, writing – original draft. Y.M.: investigation, methodology. X.D.: data collection, data analysis. L.H.: writing – review and editing, data collection. Y.G.: writing – review and editing, conceptualisation, project administration. All authors approved the final version for submission.

## Funding

This work was supported by Connotation Construction Special Nursing Advantage discipline of Nanjing Medical University (Grant 2022‐26) and Science and Technology Project of Changzhou Health Commission (Grant ZD202344).

## Ethics Statement

The experiments were conducted in agreement with the ethical standards of the ethics committee of the Third Affiliated Hospital of Nanjing Medical University ([2023]‐KY111‐01). All related patients were informed about the research and have signed the informed consent letters.

## Conflicts of Interest

The authors declare no conflicts of interest.

## Data Availability

The data that support the findings of this study are available from the corresponding author upon reasonable request, subject to ethical approvals and patient consent restrictions.
